# Atlanta Medical College

**Published:** 1866-11

**Authors:** 


### EDITORIAL AND MISCELLANEOUS.

### ATLANTA MEDICAL COLLEGE.

We have frequently taken occasion to allude to the facilities in this Institution for teaching medicine. Since the war, the building has been thoroughly repaired, and the grounds snugly fenced. In addition to this, and the replacing of destroyed apparatus, a hospital has been built in connection with the College, in which sixty to eighty beds are now constantly filled with the poor, black and white, of the city. By this means, the indigent sick of the city are cared for without expense to the city government, and a fine opportunity afforded students, during the course of lectures, to witness the treatment and progress of cases, to which they are allowed constant access. In addition to this, a Dispensary in the College has been established by the Faculty, in which fifteen to twenty indigent sick of the city, who are able to go or be carried to the College, are prescribed for daily by members of the Faculty, and medicines furnished gratuitously. These cases are examined and prescribed for, during the session, in presence of the class, and afford also an opportunity to students desiring it, for writing out and filling prescriptions.

The city authorities have thus, by a timely donation, enabled the Collge to move forward unembarrassed in the full exercise of all her departments. For this the Trustees and Faculty will ever feel grateful, and in return will endeavor to relieve the city, to the full extent in their power, from the burden of caring for the wretched poor on her hands.

The Dispensary, discontinued for a while after the close of the present year's course of lectures, is now resumed, and will be contined through the next Annual Course of Lectures, commencing the first of May next. The following statistical record shows the character of diseases, and the number of cases of each, treated in the Dispensary, from the first of May to the last of August. It will be seen that about *four hundred and thirty cases* were presented, besides received into the hospital on the College grounds during the same period:

| | |
|---|---|
| Abscess,.................2 | Ascites,..................3 |
| Anasarca,...............7 | Bronchitis,..............10 |
| Amenorrhæ,.............4 | Conjunctivitis,...........7 |
| Asthma,.................2 | Croup,...................1 |

Constipation,..............3
Cancer,..................1
Continued Fever,.........2
Caries of tibia,...........1
Cachexia,.................1
Cataract,..................1
Critical Period,...........1
Cancrum Oris,............1
Contusion,................1
Cholera Infantum,........3
Catarrh,..................4
Diarrhœa, Chronic,.......3
Diarrhœa, Acute,.........40
Duc Steno Occluded,......1
Diphtheria,...............2
Deafness,.................1
Dysmenorrhœa,...........3
Debility,..................5
Debility from Variola,....7
Debility from Rubeola,....1
Debility, Senile,..........1
Dysentery,................5
Eczema,..................1
Enteritis,.................8
Epilepsy,.................2
Elongated Uvula,.........1
Elephantiasis,.............1
Erathema,................1
Enlarged Spleen,..........1
Encisted Tumor,...........1
Enlarged Tonsils,.........1
Fistula in Ano,............1
Feruncle,.................4
Fracture of Ulner,........1
Goiter,...................2
Gonorrhœa, ..............4
Hypertrophy of Heart,....1
Hæmoptisis,..............1
Hemorrhoides,............1
Hysteria,.................11
Hepatitis,.................1
Hypertrophy of Liver,.....1
Influenza,.................2
Intermittent Fever,......23
Iritis, ....................1
Inguinal Hernia,......... 1
Irritable Uterus,..........6
Imaginary Disease,.......2
Indigestion,..............11
Irritable Stomach,........2
Inflam't'n Sub. Max.Gland, 1
Jaundice, ...............2
Lencorrhœa,.............4
Lumbago, ...............7
Laryngitis,...............1
Menengitis,...............1
Membrana Tympani dis'sed,2
Muscular contrac. of Thigh,1
Menorrhagia, ............3
Mammary abscess,........1
Marasmus, ..............1
Neuralgia,...............11
Nervous Depression,......2
Nephritis, ................1
Ovaritis, .................1
Otitis,....................1
Pleurodinia, .............1
Pertussis,................8
Pneumonia, ..............9
Prolapsus Ani,............2
Purpura,.................1
Pleurisy, ................1
Pharyngitis, ..............1
Phthisis, .................4
Phrenitis,.................1
Poisoned Wound,.........2
Phlegmasia Dolens,.......1
Prolapsus Uteri,..........1
Ptyalism, ................2
Rheumatism, Chronic,.....8
Rheumatism, Acute,.....11
Remittent Fever,.........3
Rubeola, .................1
Stomatitis, ..............8
Scabies,.................13
Strumous Ophthalmia, ....1
Spyhilis, ................22
Spinal Irritation,.........4
Sore Nipple,..............1
Scorbutus,...............2

| | |
|---|---|
| Scrofula, ................9 | Uterine Catarrh,..........1 |
| Sebaceous Glands Diseased,1 | Ulcer, ....................9 |
| Teething,................3 | Ulcerated Cornea,........1 |
| Tonsilitis, ................3 | Urticaria,.................3 |
| Torti Coli,................1 | Worms,..................17 |
| Threatened Abortion,.....1 | Whitlow,.................1 |
| Tape Worm,..............1 | Wound,...................3 |
| Torpid Liver,.............1 | Wound, Poisoned,........2 |
| Varicose Veins,..........11 | |

